# Implicating Ultrasonication and Heat–Moisture Treatments as a Green and Eco-Friendly Approach for Dual Physical Modification of *Eleocharis tuberosa* Starch to Improve Its Physico-Chemical and Functional Properties

**DOI:** 10.3390/foods14132185

**Published:** 2025-06-22

**Authors:** Zafarullah Muhammad, Rabia Ramzan, Chen Ana, Muhammad Afzaal, Adnan Abbas, Muhammad Safiullah Virk, Abdullah, Wu Sun, Guoqiang Zhang

**Affiliations:** 1 College of Biological and Food Engineering, Anhui Polytechnic University, Wuhu 241000, China; zafwahla@mail.ahpu.edu.cn (Z.M.);; 2 College of Food Science and Technology, Huazhong Agricultural University, Wuhan 430070, China; 3 Department of Food Science, Nutrition and Home Economics, Government College University, Faisalabad 5400, Pakistan; 4 School of Chemical and Environmental Engineering, Anhui Polytechnic University, Wuhu 241000, China; 5 School of Food and Biological Engineering, Jiangsu University, Zhenjiang 212013, China; 6 College of Food Science and Technology, Zhejiang University of Technology, Hangzhou 310014, China

**Keywords:** *Eleocharis tuberosa* starch, dual-physical modification, eco-friendly approach, physical and functional properties

## Abstract

Dual-physical modification is an eco-friendly and waste-free approach for enhancing the functionality of native starches compared with a single modification. In the present study, the individual and combined interrelating effects of hydrothermal (heat moisture (HM) with 15%, 20%, and 25% moisture) and non-thermal (ultrasonication (US) with 200, 400, and 600 power (W)) on the physical modification of *Eleocharis tuberosa* (Chinese water chestnut (CWCS)) starch were studied. Furthermore, their effects on the morphology, FTIR, XRD, crystallinity, thermal, pasting, swelling power, solubility, rheological characteristics, and in vitro digestibility of native and modified starches were investigated. The results indicated a consistent B-type structure of CWCS, with a significant decrease in the crystallinity (22.32 ± 0.04–28.76 ± 0.02%), which was linked with ΔH (19.65 ± 0.01–12.18 ± 0.06 Jg^−1^) and amylose content (34.67 ± 0.07–40.73 ± 0.11%). The absorbance ratio 1048/1025 specified that the combination of HM-US compacted the short-range order degree up to 1.30 for HM25–US600-CWCS. The starch treated with HM, followed by the US, considerably amplified the setback, peak, and final viscosities compared with the HM-treated starch. The rheological analysis demonstrated that the fluidity of CWCS was enhanced (G′ > G″, tan δ < 1) by the synergistic effect of HM and US, increasing the resistivity toward deformation during paste development. The dual-modified starch exhibited a slower glucose release rate with increasing moisture (25%) during HM and 600 W during the US, with higher RS contents of 45.83 ± 0.28% and 43.09 ± 0.12%, respectively. Dual-physical modification exhibited a significant aptitude for modifying native starches structurally and functionally as a substitute for product formulation with a low glycemic index.

## 1. Introduction

Finding new food sources is essential for improving food security and nutrition worldwide [1]. With the increasing global population, conventional food sources may face challenges in satisfying market demand, resulting in possible scarcity and hunger. Therefore, non-conventional and underutilized plant-based starchy foods have the potential to broaden dietary options by mitigating the burden on conventional crops and providing sustainable substitutes that are rich in vital nutrients [2]. Addressing food scarcity, enhancing health outcomes, and constructing a resilient food system for the future all depend on introducing less-exploited food sources into mainstream food systems [3].

Starch is a natural and abundant source of high-molecular-weight carbohydrates. It meets humans’ basic nutritional needs and is extensively used as a renewable resource in the industry [4]. Owing to its safety, renewability, biodegradability, abundance, cost-effectiveness, and non-texture nature, starch has gained much attention in food, pharmaceutical, and allied industries [5]. It is in tremendous demand as a raw material for producing biodegradable plastics and ethanol, as well as a thickening, bulking, gelling, stabilizing, and water-retaining agent to impart organoleptic properties to finished food products [6]. Considering the escalating demands, the starch industry continuously searches for novel sources to counteract production costs in the materials, textiles, papermaking, pharmaceutical, chemical, and food industries.

Chinese water chestnuts, scientifically known as *Eleocharis tuberosa*, belong to the Cyperaceae family and thrive in tropical climates. They feature floating leaves and round corms that develop in ponds and marshy environments [7]. They are indigenous to southern China and have a wide distribution, with 35,000 hectares covering Guangdong, Zhejiang, and Jiangsu provinces. The tubers of this plant are highly nutritious, being rich in starch and containing numerous bioactive compounds, such as cysteine, lectin, quercetin, fibers, vitamins, minerals, and essential fatty acids [8]. Chinese water chestnuts are widely consumed across Asia because of their unique, crunchy texture and potential health benefits [9]. The corm of *Eleocharis tuberosa* offers several health advantages, including antibacterial and antioxidant properties. The quality of chestnuts is believed to be closely associated with their starch properties, given their high starch content [10]. Additionally, chestnut starch holds promise as a distinctive source for food and non-food applications. Consequently, water chestnut starch is a viable alternative to conventional starch, used in several applications, and is increasingly sought by the food industry to satisfy the growing demands of the world’s population [11]. Despite the high starch content of Chinese water chestnut corms, there is still a scarcity of research on the physicochemical and functional properties of *Eleocharis tuberosa* starch. This lack of research poses a challenge in meeting the growing demand for innovative starches and in broadening the applications of Chinese water chestnut starch. Furthermore, due to the limited studies on native and modified *Eleocharis tuberosa* starch, it is critical to explore the structural characteristics of starch from *Eleocharis tuberosa* to exploit this beneficial resource in the food and allied industries [2,12].

Simultaneously, some limitations restrict the applicability of non-conventional starch independently. These limiting factors include inherent instability, lower solubility, lower light transmittance, paste clarity at room temperature, syneresis, susceptibility to thermal decomposition, and stronger retrogradation propensity [4,13]. These discrepancies can be overcome by modifying native starches using chemical, physical, or enzymatic modification methods, either alone or in combination, to enhance their functional characteristics and broaden their industrial applications [6,14]. Usually, modified starches can have better pasting properties, suitable stability, improved freeze–thaw stability, and resistance to retrogradation. As an effective fat replacer, modified starch adds one crucial application of modified starches concerning human health, which offers numerous advantages to product designers and a healthier status to customers by lowering the fat percentage in food products [15]. The selection of a modification strategy depends on the type of characteristics required in the modified starch, availability, market trends, intended application, and cost-effectiveness [16]. The most common technique for adding functional groups to starch is chemical modification, which modifies natural starch’s physicochemical and functional characteristics. Physical modification is safe, does not produce byproducts, does not pollute, is easy to manage, and does not involve chemicals. It is becoming increasingly popular as people become more conscious of the negative impacts of chemical modifications [13,17].

Based on the merits of the physical modification mentioned above, the best-fit techniques are thermal (heat–moisture treatment) and non-thermal (ultrasonic) modifications. The prospective ultrasonication treatment (UST) strategy, a promising physical modification technique, has attracted much attention [14,16]. This is because of its highly effective features, which include safety, eco-friendliness, better selectivity efficiency, fewer byproducts, and no chemical residue. It has the capacity to alter the composition, structure, and properties of starch derived from diverse sources. High-frequency sound waves (>20) are used in UST to disrupt starch particles in an aqueous medium [4]. Going through a literature review, it has been discovered that the UST impacts the physical properties of starch, such as solubility, swelling power, water absorption, gelatinization temperature, morphological features, rheological behavior, and digestibility [18,19]. Several starches, such as pea, potato, and corn starches, have been physically modified to alter their digestibility and rheological behavior using UST [20]. As a low-temperature treatment, ultrasonic processing has been integrated with various other methods to boost its efficiency and reactivity. For example, several starches have been modified using ultrasonication in conjunction with annealing, esterification, enzyme hydrolysis, γ-irradiation, and pulsed electric fields [4,20,21].

One very efficient physical method for starch modification is heat–moisture (HM) treatment. This technique is used at temperatures below the gelatinization and above the glass transition temperature (80–160 °C), with a low moisture content (<35%) [4,5]. HM influences the structural and physicochemical characteristics of starch based on various factors, including moisture content, heating time, granular composition, the organization of amylose and amylopectin, and starch source diversity [22]. Modification improves starch’s physicochemical characteristics without changing its granular structure since it keeps its rubbery mobile shape [16]. According to earlier studies, HM treatment affects the thermostability of starch structures [23], amylose concentration, relative crystallinity [24], lamellar structure, and molecular chains [25]. Furthermore, numerous studies have demonstrated that UST can modify the fine structure, gelatinization properties, chain length distribution, and rheological behaviors of starch [20,26]. Although numerous investigations have been conducted on the distinct regulation of starch via HM and US treatment, the outcomes have sometimes produced inconsistent results. For example, the taro and cassava starches’ crystalline structure did not change noticeably due to HM. In the meantime, it changed the B-type crystalline structure of potato and yam starches to an A-type one [4]. Meanwhile, UST reduced the amount of amylose in maize and pea starches [27], whereas the amylose content increased in mung bean [28], potato [27], and sweet potato [29] starches. The observed discrepancies may be ascribed to the different types of starches and conditions used for their modification. Furthermore, when native starches are modified using a combination of different techniques, their final characteristics may be altered more than when modified using a single modification method [5]. Nonetheless, the effect of this dual modification on the final characteristics of starch depends on the specific type and particular modification conditions. Recently, a study examined the impact of physical modification sequences, including ultrasonication (US), heat–moisture treatment (HMT), and their combinations, on lotus seed starch (LSS). Ultrasonication increased swelling power (17.67 g/g), solubility (17.90%), and amylose content (29.09%), whereas other treatments reduced these values. Rheological analysis showed elastic gel behavior (G′: 1665–4004 Pa > G″: 119–308 Pa), and gelatinization enthalpy increased with all treatments (16.05–17.56 J/g vs. 15.38 J/g in native). Dual modification increases resistant starch while slowly lowering digestible starch [14]. Dhull et al. (2024) [16] found that ultrasonication increased amylose content by 29.8% and water absorption by 50% in mung bean starch, whereas HMT and dual modifications reduced amylose content by 17%. The treatments decreased the oil absorption, light transmittance, swelling power, and solubility. Native starch exhibited the highest peak viscosity (5308 mPas), and the modified granules displayed surface damage. In Rice berry rice flour, wet microwave treatment yielded the highest peak viscosity (246.83 ± 7.03 RVU) and swelling power (8.98 ± 1.04 g/g), whereas ultrasound enhanced polyphenol bioavailability and reduced digestibility (eGI = 60.77 ± 0.90). Pregelatinization increases the digestibility (eGI = 69.69 ± 2.69) [30]. Luangsakul et al. (2025) [31] found that high-amylose Manpo rice had higher peak viscosity than Hommali rice. Ultrasound improves polyphenol bioaccessibility and lowers digestion rates. Acevedo et al. (2022) [22] found that HMT-UST treatment of cowpea starch enhanced thermal stability, increased resistant starch content by up to 11%, and slowed digestible starch by 30%, reducing pasting viscosity.

Based on these facts, we hypothesized that the physical modification of *Eleocharis tuberosa* (Chinese water chestnut (CWCS)) starch through ultrasonication (US), heat–moisture (HM) treatment, or a combination of both would significantly alter its physicochemical, thermal, and structural properties, making it a viable and improved alternative starch source for industrial applications. To our knowledge, the systematic physical modification of non-conventional starch from *Eleocharis tuberosa*, an underutilized variety of Chinese water chestnuts, through treatment with US and HM, is unavailable and has not yet been explored. As a result, we chose this unique variety of Chinese water chestnuts for starch as the primary material for the present investigation. The current research uses the US and HM modification methods alone and in mutual combination to thoroughly investigate and analyze their influence on modifying the shape, physiochemical characteristics, thermal stability, and short/long-range ordered structure of CWCS. Exploiting an underutilized and non-conventional starch source and an eco-friendly physical modification approach will provide new insights into expanding the industrial utilization of starch while addressing food security and sustainability in the food and non-food sectors.

## 2. Materials and Methods

### 2.1. Materials

The corms of Chinese water chestnut (*Eleocharis tuberosa*) were procured from a local market in Wuhu City, Anhui Province, China. Amyloglucosidase (99% purity, 10,000 U/mL native aspergillus niger amyloglucosidase, MW: 475.025, CAS# 9032-08-0) and α-amylase (99% pure, 50 U/mg from hog pancreas, MW: 226.232, CAS# 9001-19-8) were purchased from Shanghai Yuanye Biotechnology Co., Ltd. (Shanghai, China). The amylose (98% purity, MW: 504.437, CAS# 9005-82-07) and amylopectin (98% purity, MW: 828.718, CAS# 9037-22-03) standards were procured from Shanghai Nianxing Industrial Co., Ltd. (Shanghai, China). Milli-Q water was used to prepare solutions and emulsions. All other chemicals utilized in this investigation were of analytical grade.

### 2.2. Extraction of Starch from Eleocharis tuberosa

A procedure was followed by Muhammad et al. (2025a, 2025b) [2,12] to extract the native starch from the corms of Chinese water chestnuts (*Eleocharis tuberosa*). After being washed, peeled, and rinsed, the corms were carefully cut into small cubed pieces using a slicer. Next, the pieces were immersed in a solution of alkaline water with a pH of 9 and containing NaOH at a concentration of 1:4 (*w*/*v*) for two hours. The pieces were finely ground using distilled water (Milli-Q water) and transformed into a slurry. Next, the homogenate was filtered through 100 and 300 mm mesh sieves. The starch suspension obtained was subjected to centrifugation at 3500 rpm times for 10 min. Following removing the liquid portion, the viscous dark layer above the solid precipitate was physically scraped off. The residual pale-colored solid was rinsed twice with anhydrous absolute ethanol. Afterward, the sample was thoroughly cleaned and washed thrice with MilliQ water and centrifugated at 3500 rpm for 10 min. Subsequently, the material was dried at 30 °C for three days. Afterward, it was finely powdered and passed through 100 mesh sieves. The resulting particles were then carefully sealed in airtight polyethylene bags with zip closures and stored at −20 °C for future testing.

### 2.3. Single and Dual Modification of Eleocharis tuberosa Starch Samples

#### 2.3.1. Modification Through HM (Heat Moisture) Treatment

*Eleocharis tuberosa* (Chinese water chestnut (CWCS)) starch was subjected to modification using a slightly modified method described by Wang et al. (2018) [32]. Water was added to CWCS to change its moisture content to 15%, 20%, and 25%, which are denoted as HM15%-CWCS, HM20%-CWCS, and HM25%-CWCS, respectively. Polyethylene-sealed wet starch samples were kept at 4 °C for 48 h to achieve consistent moisture content. Afterward, wet starch samples underwent a heat treatment at 110 °C for 4 h and then dried at 40 °C for 24 h. The drying of the treated starch samples was continued till the level of moisture reached 10%, then crushed, sieved through a 100-mesh, and packed in airtight zipped polythene bags prior to experimentation.

#### 2.3.2. Modification Through Ultrasound (US) Treatment

A slightly modified approach based on Acevedo et al. (2022) [22] was used for the modification of CWCS using ultrasound treatment. To create a consistent starch suspension, the CWCS was combined with 100 mL of distilled water (5% *w*/*w*) on a magnetic stirrer and mixed for 30 min. The starch solution was then treated for 30 min via an ultrasonic processor DS-2008 (Shanghai Dusi Instrument Co., Ltd., Shanghai, China) at varying power levels (200 W, 400 W, and 600 W) and an output frequency of 50 Hz (intermittent 1 s, pulse 1 s). The energy utilization for every 100 W was 36 kJ/g. The probe of the ultrasonic processing apparatus had a tip diameter of 20 mm. During ultrasonic treatment, an ice-water bath was used to prevent temperature rise. After centrifuging the starch suspension (2654× *g*, 10 min) and drying it for 48 h at 40 °C in a hot air oven, it was strained using a 100-mesh strainer. Then, the processed starch samples were designated as US200-CWCS, US400-CWCS, and US600-CWCS, respectively.

#### 2.3.3. Dual Modification of CWCS Using Combined HM-US Treatments

The treated starch samples of CWCS with the lowest and highest moisture levels were subjected to US treatment at ultrasonic powers of 200 and 600 W to produce the HM15%-US200-CWCS, HM15%-US600-CWCS, HM25%-US200-CWCS, and HM25%-US600-CWCS samples.

### 2.4. Quantifying the Amylose Content

The K-AMYL 07/11 Amylose/Amylopectin test kit was used to quantify the AAC (apparent amylose content). Kit was procured from Megazyme International Ireland Ltd., Wicklow, Ireland. After a 5 min immersion at 100 °C in 1 mL of dimethyl sulfoxide, a 20 mg, d.b. starch sample was cooled to 40 °C and diluted to 2 mL using the kit buffer. Amylopectin was precipitated by adding 0.2 mL of concanavalin A, carefully vortexing the mixture, and then incubating it for 20 min at 40 °C. Following centrifugation at 3000× *g* for 10 min, the supernatant containing amylose was treated with 100 µL of an α-amylase/amyloglucosidase cocktail (160 U mL^−1^/330 U mL^−1^) for 30 min at 40 °C. Using a UV-mini 1240 spectrophotometer (Shimadzu), the absorbance was measured at 510 nm after glucose was released using the GOPOD reagent (0.8 mL, 20 min, 40 °C). The controls included the manufacturer’s reference starch (25% amylose) and a five-point D-glucose calibration curve (0–100 µg). The amylose percentage was determined using the formula (Glc sample/Glc total starch) × 0.9 × 100. All tests were conducted in triplicate, and the results are presented as mean ± SD.

### 2.5. Morphological Study of the CWCS Samples Using a Scanning Electron Microscope (SEM)

The starch samples were sputter coated with a thin layer of gold after being mounted on aluminum stubs using double-sided adhesive carbon tape. A HITACHI SU8600 (HITACHI, Tokyo, Japan) SEM, operating at a 5-kV accelerating voltage, was used to analyze the morphology of the starch granules.

### 2.6. Particle Size Analysis

A laser-light particle size analyzer (S3500, Microtrac Inc., Montgomeryville, PA, USA) equipped with a dry sample delivery system (Microtrac Turbotrac SDC, Microtrac Inc., USA) was used to measure the particle size distribution of the various starches. At room temperature (25 ± 2 °C), 0.5–1.0 g of dry starch powder was placed into the Turbotrac feeder and distributed into the measuring chamber using an airflow dispersion technique. According to the Mie scattering theory, the device measured the specific surface area (SSA), volume mean diameter (D[4,3]), median diameter (D[0.5]), and surface-weighted mean diameter (D[3,2]). The results are presented as mean ± standard deviation, and the measurements were conducted in triplicate.

### 2.7. Thermal Analysis of Starch Samples

A differential scanning calorimeter (DSC Q-10, TA Instruments, New Castle, DE, USA) was used to examine the thermal properties of both native and modified CWCS samples. Dried samples (3 mg) of each treatment were placed in aluminum pans and mixed with water (9 μL). The aluminum pans were then sealed with a DSC pan sealer and kept at room temperature for two hours for equilibration. The sample pans were inserted into the DSC chamber against an empty pan (reference pan) and heated from 20 to 120 °C at a rate of 10 °C/min. Afterward, enthalpy (ΔHgel) and thermal transition temperatures were automatically calculated.

### 2.8. X-Ray Diffraction

Prior to X-ray analysis, the samples were allowed to reach equilibrium for 24 h at 25 °C. Subsequently, they were carefully ground and placed into sample holders to create smooth and flat surfaces. Sample rotation was employed to reduce the orientation of the effects. Using Cu Kα radiation (λ = 0.154 nm), the X-ray diffractometer (D8 Advance, Bruker Corporation, Karlsruhe, Germany) was operated at 40 kV and 40 mA. In a glass cell, the starch powder was firmly packed and scanned at 25 °C at 5–40° 2θ angles (increments of 0.02° and a scanning speed of 2°/min) to collect diffraction data. Equation (1) was used to determine the samples’ relative crystallinity.

(1) $$Relative\;crystallinity\;\left(\%\right)=\frac{AC}{\left(AC+Aa\right)}\times 100$$

In Equation (1), where AC represents the area of the crystalline peak, and Aa denotes the area of the amorphous peak.

### 2.9. FT-IR Analyses of the Native and Modified CWCS Samples

A Vector 33 FT-IR spectrophotometer (Bruker, Ettlingen, Germany) was used to acquire the FT-IR spectra in KBr pellets. Sixty-four scans were obtained for each spectrum, with a spectral resolution of 4 cm^−1^. A meticulous sequence of steps was followed to prepare the samples for FT-IR analysis. Each sample (1 mg) was mixed with 100 mg of KBr, and the combination was finely ground in a mortar to ensure a consistent mixture. It is crucial to perform these steps quickly to reduce the chance of contamination. The sample amount must be adequate to form KBr pellets with a 4 mm diameter. Once prepared, the sample was placed in the sample compartment to acquire its spectrum. As the interferometer scans the infrared beam, a detector records the intensity of the infrared light that is either transmitted or reflected and captures it as an interferogram. The system then applies a Fourier transform to the interferogram, translating the data from the time domain into a frequency-domain spectrum, depicted as a wavenumber versus absorbance or transmittance plot. The spectrum reveals interruptions at specific wavenumbers, where distinct functional groups such as C=O, O-H, and N-H absorb infrared light. The samples were analyzed using infrared spectroscopy in the 400–4000 cm^−1^ range.

### 2.10. Measurement of the Pasting Properties

A rapid visco analyzer (TechMaster, Perten Instruments, PerkinElmer, Huddinge, Sweden) was used to test the pasting characteristics of the starch samples. The following steps were taken: 3 g of each starch (dry basis weight) was combined with 25 g of distilled water, heated to 50 °C for 1 min, and then lowered to 95 °C for 3.75 min and kept there for 2.5 min; finally, the temperature dropped to 50 °C for 3.75 min and held it for 2 min.

### 2.11. Dynamic Rheological Characteristics

The rheological behavior of the starch gels was examined using an Anton Paar MCR 302 rheometer with a 40 mm parallel plate setup and a 1 mm gap. Starch suspensions with a concentration of 5% *w*/*w* were prepared in deionized water, heated to 90 °C for 30 min to induce gelatinization, cooled to 25 °C, and allowed to equilibrate for 5 min. A strain sweep was performed to assess dynamic rheological changes in the starch paste samples. This included the measurement of the storage modulus (G′), loss modulus (G″), and loss factor (tan δ = G″/G′). The experiment was conducted at a temperature of 25 °C with a fixed strain of 2%. An angular frequency sweep was then executed, covering a range from 0.1 to 20 rad/s.

### 2.12. In Vitro Digestibility of the CWCS Starch Samples

The Babatunde et al. (2022) [26] method was used to assess the native and modified ET starch’s in vitro starch digestibility. The fractions of the starch digestion were categorized based on time of digestion: starch digested within 20 min was classified as RDS (rapidly digested starch), starch digested between 20 and 120 min was classified as SDS (slowly digested starch), and undigested starch, referred to as RS (resistant starch), was assessed based to the rate of digestion. The following equations were used to calculate the RDS, SDS, and RS:

(2) $$RDS\;\left(\%\right)=\frac{0.9\times (\rho_{20}-\rho_{0})\times V}{m}\times 100$$

(3) $$RDS\;\left(\%\right)=\frac{0.9\times (\rho_{120}-\rho_{20})\times V}{m}\times 100$$

(4) $$RS\;\left(\%\right)=100-RDS\;\left(\%\right)-SDS\;(\%)$$

### 2.13. Statistical Analyses

The analysis was performed thrice, and the data were gathered and structured accordingly. The Tukey method, utilizing Statistics 8.1 (Analytical Software, Software Version 8.1, Tallahassee, FL, USA), was applied to analyze variance (ANOVA) and mean comparisons. *p* < 0.05 was considered statistically significant. Standard errors and mean values were depicted in charts as coordinate pairs with accompanying error bars. The figures given in the manuscript were drawn using Origin Software (8.0).

## 3. Results and Discussions

### 3.1. Amylose Contents of the Native and Modified CWCS

**Figure 1:**
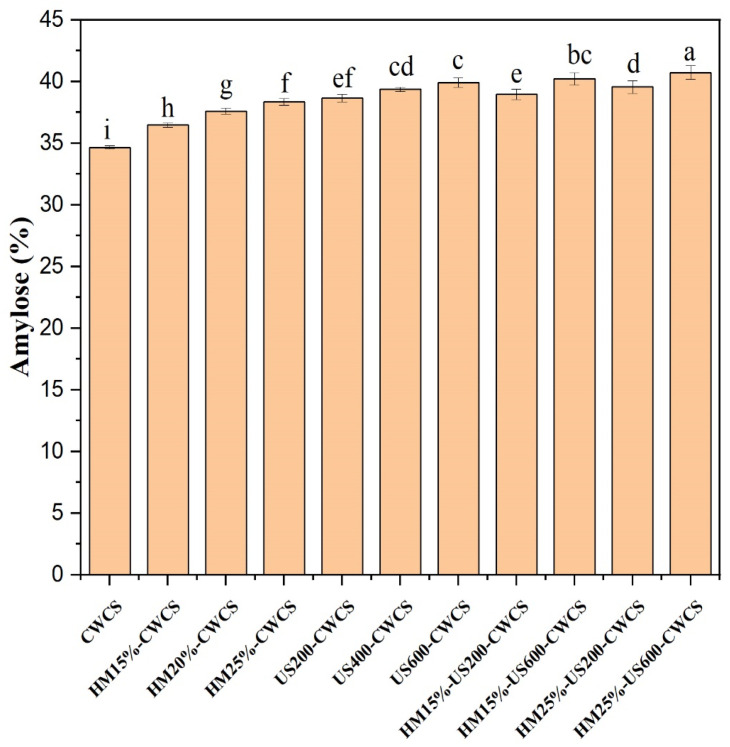
Amylose contents of the native and single-modified (HM, US) and dual-modified (HM-US) Chinese Water Chestnut Starch samples. Significant differences (*p* ≤ 0.05) exist in the mean ± standard deviation values for each sample in the same column, denoted by different superscripts.

The concentration of amylose in starch has significant repercussions for its functional characteristics. Figure 1 illustrates the results obtained from amylose profiling of the native and modified starches. Comparing native CWCS to HM treatment significantly increased the amount of amylose from 34.67% to 38.36%, respectively. This increase is probably because HM causes partial starch gelatinization at higher temperatures and the breakdown of larger starch structural molecules. As per the findings of Chen et al. (2019) [33], the HM may cause a breakdown of amylopectin molecules, leading to the formation of shorter chains similar to amylose. Nevertheless, there was no apparent variation in the amylose concentration across HM-treated samples of the starch when the moisture level rose from 15% to 25%. The amplification of the interaction between amylopectin, amylose, and lipid molecules may modify the amylose concentration.

Furthermore, during HM treatment, the hydrogen bonds between co-crystallized amylose and amylopectin may be broken, and amylose’s iodine binding abilities might increase, enhancing the apparent amylose contents (AAC) [34]. Ultrasonication (US) treatment further elevated the amylose concentration in starch particles, and these effects were substantially (*p* < 0.05) correlated with the power of ultrasound up to 39.91% (US600-CWCS).

This result may be associated with the partial depolymerization of the chains caused by the mechanical stress from ultrasonic treatment. This resulted in an improved amylose content due to the increased linear fragments in the modified starch samples [19]. Furthermore, ultrasonication following heat–moisture treatment was more effective than heat–moisture treatment alone in increasing amylose content up to 40.73% (HM25%-US600-CWCS) and 40.23% (HM15%-US600-CWCS). This suggests that heat energy enhanced the mobility of starch molecules, rendering them more susceptible to subsequent ultrasonication degradation. Earlier studies have shown that heat–moisture treatment generates pressure and heat, inducing water molecule penetration into starch granules and eventually disrupting their hydrogen bonding [35]. This modification lessens the aggregation in the crystalline region of amylopectin within starch particles and heightens their susceptibility to ultrasound (US) treatment. These findings align with those of Su et al. (2024) [4], who assessed the impact of ultrasound on modifying purple rice starch.

### 3.2. Morphology and SEM Analysis

**Figure 2:**
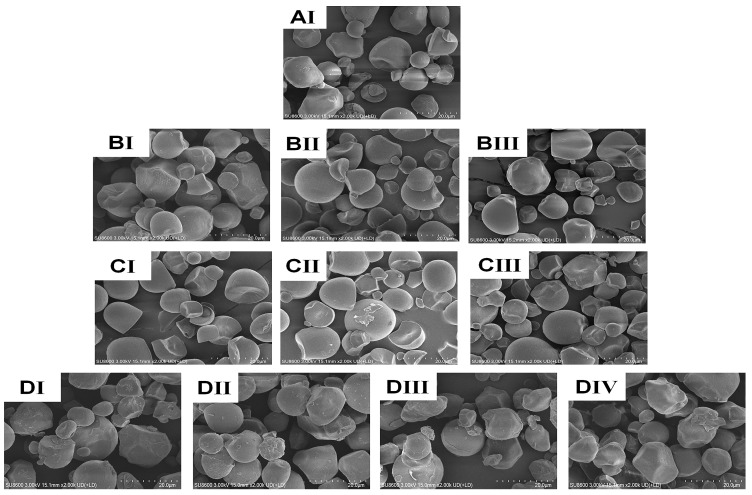
SEM images of native and the single modified (HM, US), dual modified (HM-US) Chinese Water Chestnut Starch samples. (magnification: 2000×). (**AI**) native CWCS; (**BI**–**BIII**) correspond to the HM15%-CWCS, HM20%-CWCS, HM25%-CWCS samples, respectively; (**CI**–**CIII**) correspond to the US200-CWCS, US400-CWCS, US600-CWCS, respectively; (**DI**–**DIV**) correspond to the HM15%-US200-CWCS, HM15%-US600-CWCS, HM25%-US200-CWCS, HM25%-US600-CWCS, respectively.

The shape, size, and surface characteristics of granules are crucial for the use of both dietary and non-food starches. In Figure 2, scanning electron microscope (SEM) pictures of both native and treated CWCS granules are presented, illustrating the impact of the heating (HM) and ultrasonic (US) treatment. The granules in the native sample (Figure 2AI) have a smooth, regular surface and are spherical or polygonal. Nevertheless, granule size, aggregation, surface roughness, and dent formation of starch granules all increased in single-modified HM (Figure 2BI–BIII). HM generated aggregation phenomena and concavity in the starch granules, and the degree of these changes increased with higher moisture levels. The HM samples with higher moisture content noted more significant aggregation and surface fusion levels of the granules [5]. This phenomenon may be attributed to the accumulation of high pressure inside the starch granules, resulting in their significant enlargement. Given that the granules were unable to withstand this rapid expansion through hydration, the starch molecules processed by HM burst or collapsed instead of swelling [14]. As the ultrasonic impact varied (Figure 2CI–CIII), the starch granules underwent plication, scragginess, disintegration, and fissures on their surface after processing. Additionally, the porosity in these granules becomes more apparent compared with the original starch granules. The US treatment process can cause mechanical damage to starch granules by causing the collapse of cavitation bubbles. This collapse generates high local velocities of the liquid layers around the starch granules owing to the immense pressure gradients. Consequently, the starch granules rupture and undergo mechanical damage. Ultimately, the generation of shear force can lead to the formation of holes and fractures within the granular structure. The outcomes of earlier research on sonicated starches are consistent with our findings [36].

The starch particles that underwent HM-US treatment exhibited dispersion of the originally agglomerated granules, as well as visible flaws such as fissured and coarser surfaces, dents, and even rupturing of certain starch granules on their exterior surfaces, as shown in Figure 2DI–DIV. These findings suggest that starch granules treated with Heat moisture (HM) treatment are more prone to ultrasonication (US) compared with untreated, native starch granules. This is because starch polymer chains become more mobile in the presence of moisture and heat, making amylose easier to leach and changing the radial configuration of the amylopectin crystals [37]. Therefore, this feature may be attributed to the shear cavitation process induced by microbubbles during ultrasonication (US), which is enhanced by partial gelatinization during HM. Moreover, the ultrasonic process generates free radicals (°OH and °H), which first induce the fragmentation of starch chains and subsequently disturb the internal molecular arrangement [38]. Integration of these two modifications expands the potential interactions between starch and other molecules, such as proteins and bioactive substances.

### 3.3. Particle Size Distribution of Native, Single, and Dual Modified Starches

To assess the impact of single treatment HM, US, and dual HM-US on CWCS granules, particle size distribution measurements were presented as D[2,3], D[4,3], and D[0.5] in Table 1. The values of D[2,3], D[4,3], D[0.5], and SSA values of native CWCS were 5.78 ± 0.08 μm, 12.95 ± 0.05 μm, 9.84 ± 0.03 μm, and 1.08 m^2^/g, respectively.

After HM, CWCS particles tended to gradually become bigger in size, with the increase getting more noticeable as the moisture content increased HM15%-CWCS < HM20%-CWCS < HM12%-CWCS. This observation supports the SEM (Figure 2) results by indicating that HM caused the starch granules to aggregate. Compared to native CWCS, there was a slight variation in the samples subjected to US at different power levels: US200-CWCS > US400-CWCS > US600-CWCS. The exfoliation of starch granules was ascribed to ultrasonication’s mechanical and cavitation effects [39].

The findings demonstrate that the US caused changes in the surface and microstructure of granules, affecting the integral structural stability of the starch particles at a minimal level. Furthermore, compared with single HM and US treatments, a significant increase in the size of the particle was seen when the starch samples received the combination HM-US treatments. For instance, HM15%-US-200-CWCS, HM25%-US-200-CWCS D[3,2], and D[4,3] were less than HM15%-US-600-CWCS and HM25%-US-600-CWCS’s, indicating that high power ultrasound cause cavitation and shear forces contributed to the granules’ spreading. Furthermore, Table 1 shows that the SSA values of HM and US starch particles with varying percentages of moisture decreased from 0.89 ± 0.02 to 0.83 ± 0.01 m^2^/g and 1.05 ± 0.01 to 0.98 ± 0.02 m^2^/g, respectively. It has been reported that the thermal and rheological characteristics of the starch particles were affected by SSA, which improved their water absorption. Subsequently, the starch paste became more fluid as the moisture content in HM increased, particle size expanded, and SSA decreased [40]. The cavitation forces generated by ultrasound enhance the permeability of starch granules to water during heating. In contrast to HM alone, dual HM-US decreased fluidity as starch granules increased and SSA decreased. This result corresponds to the reported modifications in the rheological characteristics. Furthermore, it was shown that both single (HM, US) and dual (HM-US) alterations changed the size of the starch particles. This ultimately affects the digestibility of starch by influencing the diffusion and adsorption of enzymes [4].

### 3.4. Thermal Characteristics of Starch Samples

According to the DSC investigations, physical modification modifies the native starch’s thermal characteristics. Table 2 shows the gelatinization transition temperatures, which encompass the onset (T_o_, peak (T_p_), and conclusion (T_c_) temperatures, as well as the respective enthalpy of gelatinization (ΔH), gelatinization range (T_r_), and peak height index (PHI). The T**_o_**, T_p_, and T_c_ temperatures of the CWCS were recorded as 58.08 ± 0.22 °C, 67.32 ± 0.41 °C, and 79.14 ± 0.66 °C (Table 2).

At the same time, modification through different treatments, such as HM, US, and HM-US, increases T**_o_** up to 61.72 ± 0.95 °C, 59.29 ± 0.25 °C, and 60.68 ± 0.54 °C (Table 2), T_p_ up to 70.23 ± 0.74 °C, 68.64 ± 0.05 °C, and 69.43 ± 0.13 °C (Table 2), and T_c_ up to 84.75 ± 0.26 °C, 80.72 ± 0.85 °C, and 82.89 ± 0.94 °C (Table 2), respectively. The increase in T**_o_**, T_p_, and T_c_ has been ascribed to changes in the structure of starch granules, such as connections between lipids and amylose, inhibiting the mobility of starch chains within the amorphous lamellae [41]. As the gelation temperature rose, ΔH decreased HM-UT < UT < HM < CWCS (Table 2); this effect intensified with the increase in moisture content during the HM and power level of US. In dual HM-US-treated starch, the corresponding values were noted to be 12.18 ± 0.25 Jg^−1^ (HM25%-US600-CWCS) and 14.64 ± 0.31 Jg^−1^ (HM15%-US600-CWCS), respectively. The natural CWCS’s gelatinization temperature range (T_r_), ΔH, and PHI values were 21.06 ± 0.32 °C, 19.65 ± 0.41 Jg^−1^, and 2.12 ± 0.01 J g^−1^, respectively.

By enhancing the helical arrangement’s mobility and breaking the hydrogen bonds between double helices, HM lowers the overall ΔH [42]. However, when the ultrasonic power increased, ΔH dropped, suggesting a partial transition to an amorphous structure from a semi-crystalline one. Furthermore, after HM was coupled with the US, ΔH decreased even further, most likely because the semi-crystalline portion was more prone to US-induced disintegration after HM. US treatment produced a shear cavitation effect that led to surface pitting and cracking, which improved water transport to the starch granules during gelatinization [4]. The PHI shows the gelatinization uniformity and the ratio of ΔH to the gelatinization temperature range [26].

**Figure 3:**
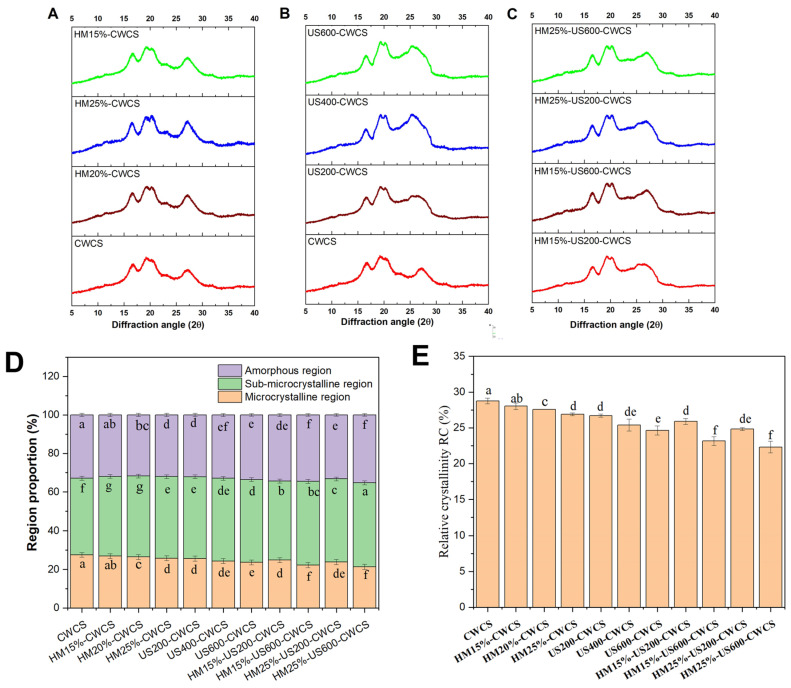
XRD patterns ((**A**) (HM), (**B**) (US), (**C**) (HM-US)), proportion of different region types (**D**), relative crystallinity RC (**E**), for the native and the single modified (HM, US), dual modified (HM-US) Chinese Water Chestnut Starch samples. There are significant differences (*p* ≤ 0.05) in the mean ± standard deviation values for each sample in the same column, denoted by different superscripts.

The XRD study showed (Figure 3) that the HM affects the crystalline perfection. The results from the XRD corroborated the DSC findings, providing mutual confirmation. They demonstrated how ultrasound decreased the order of the starch crystalline structure and double helix crystals. The generation of additional short-chain amylose through the localized cleavage of amylopectin branches increases the structural disorder, thereby decreasing ΔH and promoting surface roughening, which aligns with the findings from the SEM analyses (Figure 2). The results indicate that the highest amylose formation in HM, followed by US-modified samples, correlates with the most substantial decrease in ΔH. Thus, the research substantiates that the elevation of short-chain amylose may result in complications in HM-US. The pronounced impact of HM and US under optimum conditions showed significantly elevated gelatinization temperature and reduced enthalpy compared with other treatments.

### 3.5. Crystalline Structure Using XRD

X-ray diffraction (XRD) patterns may offer qualitative evidence for the formation of V-type inclusion complexes. Figure 3A–C displays the diffraction patterns, and Figure 3E represents the relative crystallinity (RC) values for natural, single-, and dual-modified starches, respectively. The native CWCS starch displayed a significant diffraction peak at about 17° 2θ, along with several smaller peaks near 19°, 20°, and 27° 2θ (Figure 3A–C). The peak at approximately 17° 2θ suggests a B-type crystallinity. In contrast, the peak at 20° 2θ indicates an A-type crystallinity.

The modified starch samples showed no extra diffraction peaks at other locations, suggesting that the preexisting crystalline shape was preserved and no new crystalline structures were generated. The findings indicated that HM treatment and the US did not alter the crystalline pattern of CWCS, corroborating previous studies. However, there was a significant reduction in the intensity of the four diffraction peaks in the XRD patterns compared with that of native starch, particularly with increasing sonication strength. Likewise, some studies have reported that dual modifications of HM followed by US did not affect the crystalline structure, as observed in pea and cowpea starches [17,22].

Compared with their unmodified counterparts, the relative crystallinity of CWC starch granules was significantly high (28.76%, *p* < 0.05) for all single- and dual-modified starches, representing the following pattern CWCS > HM > US > HM-US (Figure 3E). The results show that the crystalline structure of CWCS was damaged by all the treatments used in this investigation. This decrease in the relative crystallinity of HM starch granules may have been caused by either an increase in the amorphous region within the semi-crystalline lamella or a decrease in crystallinity. The disintegration of amylopectin crystals and the weakening of the layered structure in starch granules that have undergone heat–moisture treatment decrease relative crystallinity [43].

The degree of damage to the crystal structure correlates with its crystal size, original crystallinity, crystal size, and helical structures. Moreover, the combined impact of heat and moisture may lead to the unwinding of the spiral ends of the swollen starch particles, expediting the displacement and reorganization of the molecular chains into a more disorganized configuration [44]. Furthermore, an increase in ultrasonic power led to a decrease in the RC of the starch samples that underwent ultrasonic treatment. The findings indicate that ultrasonication may reduce the relative crystallinity of starch. This happens as a consequence of cavitation and mechanical oscillation pressure during ultrasonic irradiation, which breaks down hydrogen bonds and starch chain structures. These factors damage the loosely packed lattices and amorphous regions and modify the orientation of the double-helix structures, ultimately resulting in a decline in starch crystallinity [45]. The relative crystallinity (RC) of the starch granules decreases significantly (Figure 3E) in dual modification HM followed by ultrasonication (US) treatment compared with starches treated solely with HM under identical conditions. These findings align with the enthalpy changes (ΔH) measured by DSC (Table 2). The reduction in RC may be attributed to the deformation of the double helices within the crystalline regions induced by the combined effects of HM and US. It has been observed that HM-modified starch, when subsequently subjected to US, exhibits increased crystallite heterogeneity, which contributes to the decreased RC [46].

The percentage of microcrystalline areas dropped by around 6% in the single and dual-modified starches group, as seen in Figure 3D. In comparison to CWCS, the percentage of the amorphous regions rose from 32.81 ± 0.08% to 33.22 ± 0.07%. The post-modification instability of the multilayered arrangements of the modified starch may explain this observation. Compared with the US and HM, which relied on DSC and RC results, the dual-modified group had a more significant percentage of amorphous areas. This result suggests that the US HM alteration deconvoluted the double helix of the starch, and the starch molecular chain was broken.

### 3.6. FTIR

**Figure 4:**
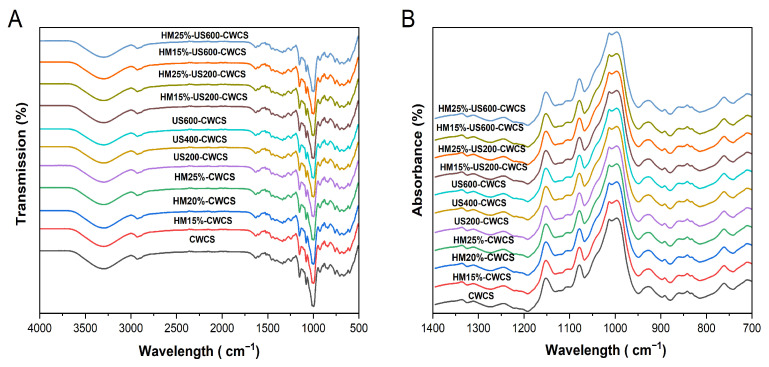
FTIR spectra (**A**,**B**) of the native and the single-modified (HM, US) and dual-modified (HM-US) Chinese Water Chestnut Starch samples.

Figure 4 displays the FT-IR spectra of the native, single-modified HM15%-CWCS, HM20%-CWCS, HM25%-CWCS, US200-CWCS, US400-CWCS, US600-CWCS, and dual-modified HM15%-US200-CWCS, HM15%-US600-CWCS, HM25%-US200-CWCS, and HM25%-US600-CWCS.

Natural CWC starch’s functional groups remained unchanged under these treatments, indicating that although the amplitudes of the peaks varied, neither the single (HM/US) nor dual (HM-US) alterations produced new functional groups or chemical bonds. In the hydroxyl group, the -OH stretching is represented by broad stretching with a center at 3000–3600 cm^−1^. There was no shift in this band in any of the modified CWCS samples, suggesting that the hydroxyl group in the starch remained unchanged [47]. The principal absorption peak at 2940 cm^−1^ signifies a typical polysaccharide, while the intensity bands observed in the 2800–3000 cm^−1^ region result from the asymmetric stretching of the -CH bond. Due to the hydroxyl group’s deformation vibration in water, an absorption peak was seen at 1637 cm^−1^ (Figure 4).

The vibration of the C–O–C glycosidic bond caused the formation of weak bands in the 1196–1529 cm^−1^ range. The bending vibrations of O–H and C–H from adsorbed water are associated with sharp bands in the 1523–1648 cm^−1^ range. The most noticeable peak, located at 1005 cm^−1^, is sensitive to variations in crystallinity and may be linked to the vibration of C–O–H and the amorphous state of the starch [20]. Concerning the hydrogen bonds of the anhydrous glucose unit, the absorption peaks at 918 cm^−1^ may be attributed to the skeletal mode vibration of the α-(1,4) glycosidic linkage. These findings were congruent with prior research [41].

The absorbance band shows the helical structure of starch at 995 cm^−1^ caused by the C–OH bending vibrations. The bands at 1048 and 1025 cm^−1^ correspond to starch’s crystalline and amorphous forms, respectively. The deconvoluted FTIR spectrum’s absorbance ratios of 1025/995 and 1048/1025, and Figure 4B may be used as indicators to determine the degree of order to describe changes in the short-range ordered structure and the interactions among the molecular chains of the starch [41,48]. The internal modifications in the double helix degree are indicated by the R1025/995 ratio. Table 3 shows the data for various starch samples.

While the 1025/995 values rose from 0.52 to 0.71, the 1048/1025 values of HM starches dropped from 1.60 to 1.38 with increasing moisture contents (15, 20, 25%) compared with native CWCS. These modifications imply that the HM has disrupted the internal double helix degree and the short-range ordered structure. These findings could be attributed to the partial gelatinization of the starch samples, which can disturb the amylopectin’s ordered crystalline structure [49]. The degree of molecular structure that is short-range ordered was decreased due to the unwinding or irregular packing of helical structures of the starch caused by the rapid movement of water molecules during thermal processing [50,51]. In comparison to the native starch, US starches showed somewhat lower R 1048/1025 values; when the ultrasonic power rose to 600 W, this ratio dramatically decreased to 1.47. The results suggest that ultrasound treatment primarily disrupted the amorphous regions of starch particles while exerting nominal effects on the crystalline assemblage, likely due to the explicit conditions of the US application. According to reports, the crystalline zones of starch particles are less vulnerable to ultrasonic damage than the amorphous areas [52]. Even so, the R 1048/1025 for HM25%-US600-CWCS is 1.30, which is still less than that of the original starch, considering the hybrid or dually modified (HM-US) starch samples. This indicates improved short-range order ascribed to better starch chain interactions and encouraging a comparatively ordered structure by subjecting it to ultrasonication.

### 3.7. The Pasting Characteristics of the Native Single and Dual Modified CWCS Starches

**Figure 5:**
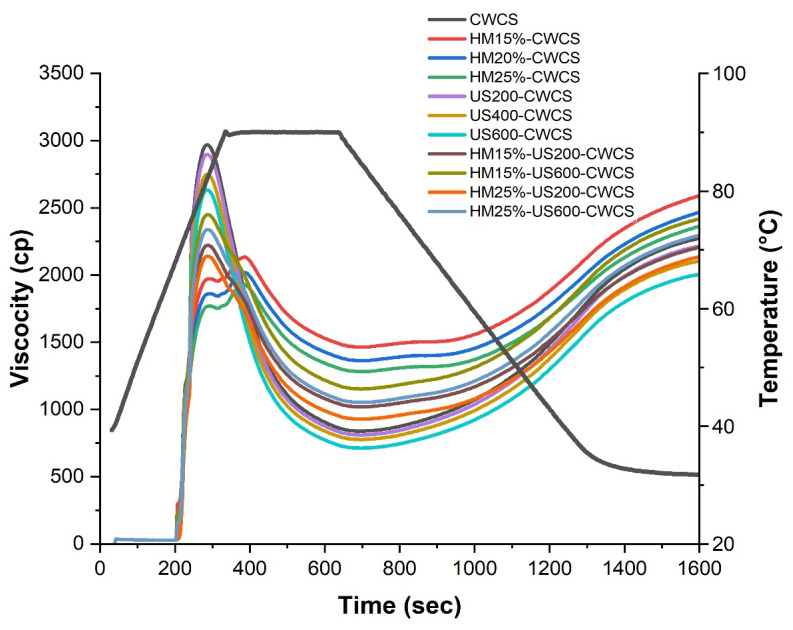
RVA curves of native and the single-modified (HM, US) and dual-modified (HM-US) Chinese Water Chestnut Starch samples.

As can be seen, Figure 5 presents the pasting properties of both native and modified CWCS starches. Compared with the native CWCS starch, a highly significant decrease (*p* ≤ 0.01) was observed in all viscosity parameters of single HM-modified samples (HM15%-CWCS, HM20%-CWCS, HM25%-CWCS), as well as a significant decrease (*p* ≤ 0.05) in US200-CWCS, US400-CWCS, US600-CWCS.

These parameters include the peak, trough, breakdown, final, and setback viscosities. Amylose content has a crucial link with the viscosity characteristics of the starch [17]. However, the pasting profile of CWCS starch changed when it was subjected to HM (15, 20, and 25% moisture), and a lower breakdown and final and peak viscosities were obtained. The viscosity decreased due to the swollen starch granules being subjected to shear stress and breaking apart during the high-temperature holding phase. This phenomenal behavior intensified with the increasing moisture content during HM treatment [53]. These changes could be induced during HM due to structural rearrangements resulting from changing the starch-chain linkages and crystalline orders to the amorphous regions of the starch molecules. XRD results (Figure 4) confirmed that HM could induce interactions between the lipid and starch molecules. These interactions can restrain the leaching of amylose during the heating of the starch suspensions [54]. Regarding dual modifications, the effects of HM-US on the pasting characteristics of starches treated at varying moisture levels (15% and 25%) and wave power (200 W and 600 W) varied significantly (*p* < 0.05), particularly on the peak, trough, breakdown, and final viscosities. Compared with the starch samples that underwent single HM or HM15%-US600-CWCS and HM25%-US600-CWCS treatments (Figure 5), samples showed higher peaks, troughs, and final viscosities. The maximal swelling capacity of starch granules is indicated by the peak viscosity. The increased trough and peak viscosities suggested that the starches were more resistant to heat and shear. This could be because US-induced amylopectin depolymerization improved the rearrangement of starch chains and the interaction between starch chains and lipids, which improved the stability of the amorphous areas in starch granules. The capacity of starch pastes to withstand high temperatures is represented by their breakdown. This phenomenon can primarily be attributed to the reinforcement of the amorphous region within starch granules during HM treatment, followed by US, leading to swollen starch granules exhibiting notable resistance to disintegration. Because amylose molecules aggregate throughout the cooling process, the final viscosity indicates an increase in the viscosity of the starch paste.

### 3.8. The Dynamic Rheological Characteristics

**Figure 6:**
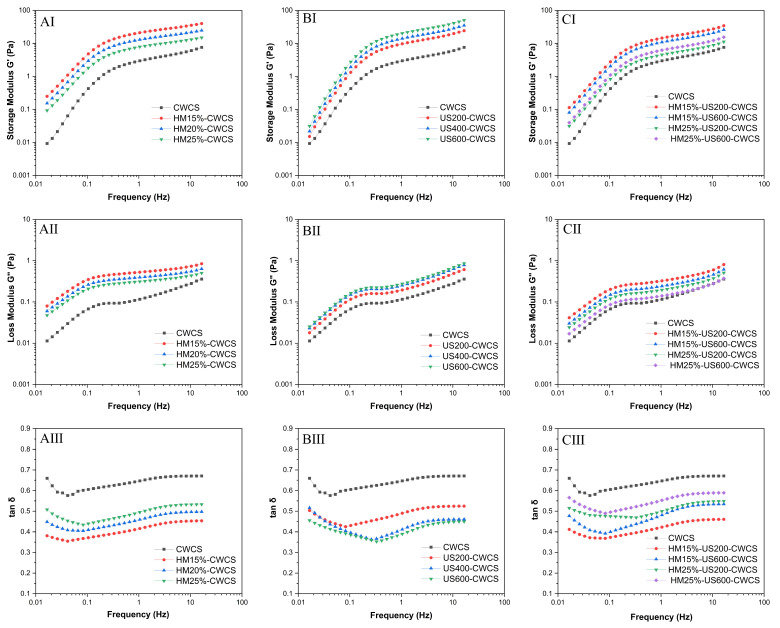
(**AI**–**AIII**,**BI**–**BIII**,**CI**–**CIII**) Dynamic frequency sweep (G′, G, and tan δ) of the native and single modified (HM, US) and dual-modified (HM-US) Chinese Water Chestnut Starch pastes (5%, *w*/*w*).

A minor deformation oscillatory frequency sweep was used to examine the dynamic viscoelastic characteristics (Figure 6). The storage modulus (G′) represents the elasticity of the fluids (Figure 6AI,BI,CI), while the loss modulus (G″) indicates the viscosity (Figure 6 AII,BII,CII). The tan δ = G″/G the formula used to compute the loss tangent. Throughout the entire frequency spectrum, G′ exceeded G″ (tan δ < 1).

Both G′ and G″ moduli for all samples demonstrated an increase with rising frequency, indicating a characteristic weak gel structure [55]. For all starch samples, tan δ showed a dependency on angular frequency (ω), as shown in Figure 6AIII,BIII,CIII, highlighting the superiority of the elastic behavior over the viscous behavior, whereas the reverse behavior was observed for HM treatment. Compared with the native CWCS (more liquid-like sample), both single HM15%-CWCS, HM20%-CWCS, HM25%-CWCS, US200-CWCS, US400-CWCS, US600-CWCS, and dual modifications (HM15%-US200-CWCS, HM15%-US600-CWCS, HM25%-US200-CWCS, HM25%-US600-CWCS) showed lower tan δ values, suggesting that deformation was practically reversible and that the starch gel was stiff. They also exhibited a considerable ability to store water. The single modification, such as HM and US, resulted in G′ values greater than those compared with the native paste, suggesting that the CWCS paste may withstand deformation better via physical modification. According to reports, starch molecules, particularly amylose, end up re-forming double helices, which causes granules to degrade due to cavitation pressures. Consequently, the granules became more accessible to water, increasing their elasticity and producing a stiffer gel with a higher G′. The breakdown of starch structures due to US treatment and the subsequent development of viscoelastic crested polymer chains from starch retrogradation may be the cause of the elevated G′ and G″ values and lower tan δ values [56]. However, the G′ values of HM25%-US600-CWCS were lower than those of HM15-US600-CWCS, indicating a modest decrease in deformation resistance following the combined modification (HM-US). It is possible that the melting of the crystalline area, which softened the starch granules, produced the drop in G′ values. This could be explained by the fact that US treatment caused the starch granules to sustain significant damage from shear stress. Consequently, amylose molecules are straightened, and the effect of shear action within the fluid layers diminishes, reducing viscosity and weakening the gel network structure [57]. HM produced short amylose chains via localized breaking down of amylopectin branches, resulting in an elevation of G′. G′ may rise as a result of the lateral interchain interactions being initially favored by the highest concentration of shorter amylose chains. However, the application of the US to HM-treated samples accelerated the process and prevented the formation of a cohesive, rigid network throughout gelatinization [24]. Thus, single or dual modification may affect starch structure and thermal stability, which can change the starch’s gel and gelatinization characteristics.

### 3.9. In Vitro Digestion

**Figure 7:**
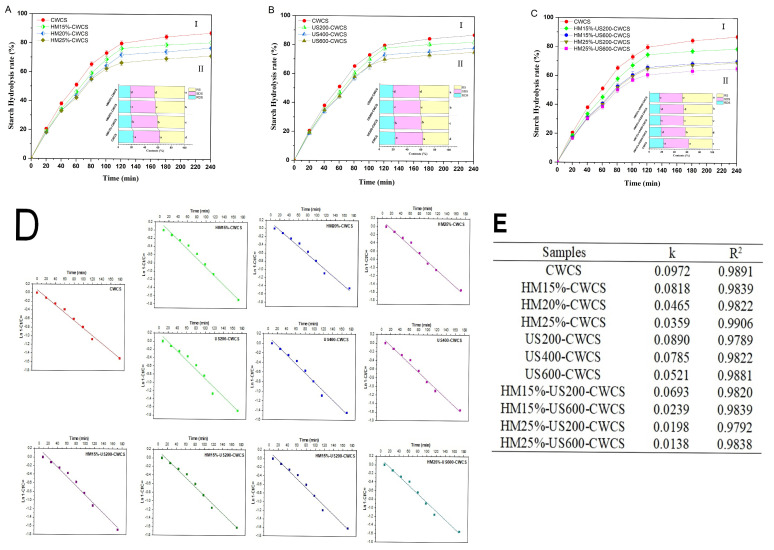
Starch hydrolysis curve ((**A**(I)) (HM), (**B**(I)) (US), (**C**(I)) (HM-US)), digestibility fractions ((**A**(II)) (HM), (**B**(II)) (US), (**C**(II)) (HM-US)), hydrolysis fitting curve (**D**), and hydrolysis parameters (**E**) of native and single modified (HM, US), dual modified (HM-US) Chinese Water Chestnut Starch samples. There are significant differences (*p* ≤ 0.05) in the mean ± standard deviation values for each sample in the same column, denoted by different superscripts.

The digestion profiles and plots for the logarithm of slope (LOS) and its parameters for each starch sample are shown in Figure 7. The in vitro digestion rate of single and dual modified starches is presented in Figure 7A(I),B(I),C(I), while these starches’ RDS, SDS, and RS content are displayed in Figure 7A(II),B(II),C(II). The digestibility was reduced due to the lower RDS content in HM, US, and dual-modified HM-US treated starches compared with native starch (Figure 7A(I),B(I),C(I)). The RDS substantially decreased (*p* < 0.05) when HM moisture levels rose 15–25%. On the other hand, HM-treated samples had a higher RS content than native starch, and this value rose significantly (*p* < 0.05) as treatment moisture increased (Figure 7A(II)).

This result showed that HM treatment was a valuable technique for controlling CWCS starch’s digesting qualities. The findings may be ascribed to HM facilitating interactions among starch chains and creating starch–lipid complexes, which exhibited reduced susceptibility to digestive enzymes. The RS contents rose from 37.23 ± 0.06% to 45.83 ± 0.28%, respectively, whereas the RDS content considerably dropped from 22.67 ± 0.52% to 17.50 ± 0.43%. These results correspond to a previous study on corn starches [58], which demonstrated that the amylopectin structure and interactions developed during HM treatment significantly influence the contents of RDS, SDS, and RS starches.

Likewise, whereas US treatment decreased the RDS highly significantly (*p* < 0.01) and SDS significantly (*p* < 0.05) concentration in starches, it raised the RS (37.23 ± 0.06% to 43.09 ± 0.12% (Figure 7BII). According to Sullivan et al. (2018) [59], US treatment may cause starch chain depolymerization and reduce the connections between starch and proteins, increasing starch hydrolysis. The RS content of the US-treated starch was higher than that of the native starch owing to the formation of double helices, as confirmed by FT-IR. This, in turn, made the crystalline regions more compact and resistant to digestion by enzymes [35]. Moreover, among the dual-modified starches, HM25%-US600-CWCS substantially (*p* < 0.01) enhanced the RS content by 47.96 ± 0.154%. Therefore, combining the two modifications will help make the modified starch more digestion-resistant (Figure 7CII). The hydrolysis kinetics LOS plots and fitted curves for each starch sample are shown in Figure 7D, respectively. Figure 7E summarizes the digestion rate coefficients (k) and coefficient values (R^2^) derived from these plots. The slowest digestion was indicated by HM25%-US600-CWCS, which had the lowest digestion rate (k), which measures the reaction rate in the LOS model for amylolysis. With R^2^ higher than 0.9789, the hydrolysis kinetic models and hydrolysis curves fitted well, and the k value showed the rate of enzymatic digestion. The k value of CWCS increased following HM, US, and dual HM-US treatment, indicating improved sensitivity to amylose hydrolysis and an enhanced degree of starch digestibility.

## 4. Conclusions

In the present research work, physical modification of the native starch from an underutilized variety of Chinese water chestnut (*Eleocharis tuberosa*) was carried out to assess the impacts of single (HM or US) and hybrid or dual modifications on its rheological, pasting, digestibility, and multi-scale structural properties. HM caused partial gelatinization and granule expansion, increasing granule aggregation and particle size as the moisture content increased. There might be a decrease in short and long-range degrees of the starch molecules due to the destabilized lamellar array and destruction of the amylopectin crystallites owing to the impact of HM at different moisture levels. Furthermore, US treatment with the increase in the power (200, 400, 600) level significantly altered the properties of starch, such as the surface of granules and internal structure, without disturbing the internal integrity of starch particles. However, the combination of low and high moisture levels of HM, followed by minimum and maximum power of US, intensified the instability of the crystal structure of HM samples. The dual modification enhanced the gelatinization of starch and improved its fluidity. The characteristics of CWCS may thus be considerably modified by the synergistic modification of HM-US, which may be helpful as an environmentally friendly product that produces no byproducts. After single and dual modification, rheological and textural analysis showed that the double helix structure of CWCS was damaged and that the crystal structure formation was prevented. As a result, the starch’s gelatinization enthalpy decreased. Using these attributes may change the viscosity and texture of food products based on starch; for example, the texture and cooking characteristics of pasta and noodles can be improved. Also, dual-modified starch enhanced thermal stability, indigestibility, and gel stability while protecting its multi-scale structure. According to this study, a combination of HM and US modification may produce starch with particular qualities, which has the potential to formulate a variety of products using under-utilized non-conventional sources.

## Figures and Tables

**Table 1:** Particle size distribution data of the native and single modified (HM, US) and dual modified (HM-US) Chinese Water Chestnut Starch.

Starch Type	D[2,3]	D[4,3]	D[0.5]	SSA
	µm	µm	µm	m^2^/g
CWCS	5.78 ± 0.08 ^ij^	12.95 ± 0.05 ^k^	9.84 ± 0.03 ^k^	1.08 ± 0.01 ^a^
HM15%-CWCS	6.75 ± 0.01 ^f^	19.19 ± 0.08 ^g^	15.15 ± 0.16 ^g^	0.89 ± 0.02 ^f^
HM20%-CWCS	6.96 ± 0.04 ^e^	21.27 ± 0.04 ^e^	17.51 ± 0.09^3^	0.86 ± 0.00 ^g^
HM25%-CWCS	7.20 ± 0.12 ^d^	23.64 ± 0.17 ^d^	19.76 ± 0.02 ^d^	0.83 ± 0.03 ^g h^
US200-CWCS	5.85 ± 0.02 ^i^	13.42 ± 0.01 ^j^	10.08 ± 0.01 ^j^	1.05 ± 0.01 ^b^
US400-CWCS	5.97 ± 0.01 ^h^	13.90 ± 0.09 ^i^	10.82 ± 0.02 ^i^	1.02 ± 0.02 ^c^
US600-CWCS	6.09 ± 0.16 ^g^	14.79 ± 0.02 ^h^	10.93 ± 0.04 ^h^	0.98 ± 0.02 ^d^
HM15%-US200-CWCS	6.90 ± 0.09 ^e^	20.66 ± 0.05 ^f^	16.78 ± 0.06 ^f^	0.87 ± 0.01 ^g^
HM15%-US600-CWCS	7.83 ± 0.02 ^b^	25.91 ± 0.01 ^b^	20.61 ± 0.07 ^b^	0.76 ± 0.01 ^i^
HM25%-US200-CWCS	7.56 ± 0.05 ^c^	25.08 ± 0.13 ^c^	20.35 ± 0.01 ^c^	0.80 ± 0.00 ^h^
HM25%-US600-CWCS	8.04 ± 0.01 ^a^	26.16 ± 0.11 ^a^	21.38 ± 0.08 ^a^	0.69 ± 0.01 ^j^

There are significant differences (*p* ≤ 0.05) in the mean ± standard deviation values for each sample in the same column, denoted by different superscripts on the shoulders of the same line.

**Table 2:** Thermal (DSC) properties of the native and single-modified (HM, US) and dual-modified (HM-US) Chinese Water Chestnut Starch.

Starch Type	T_o_ (°C)	T_p_ (°C)	T_c_ (°C)	ΔH ((J/g)	T_r_ (°C)	PHI (Jg^−1^ °C^−1^)
CWCS	58.08 ± 0.22 ^g^	67.32 ± 0.41 ^fg^	79.14 ± 0.66 ^h^	19.65 ± 0.41 ^a^	21.06 ± 0.32 ^a^	2.12 ± 0.01 ^a^
HM15%-CWCS	59.91 ± 0.11 ^c^	69.24 ± 0.25 ^c^	82.37 ± 0.72 ^d^	18.86 ± 0.39 ^b^	22.46 ± 0.71 ^a^	2.03 ± 0.01 ^a^
HM20%-CWCS	60.75 ± 0.75 ^ab^	69.65 ± 0.09 ^ab^	83.59 ± 0.14 ^b^	17.43 ± 0.16 ^d^	22.84 ± 0.96 ^a^	1.95 ± 0.06 ^b^
HM25%-CWCS	61.72 ± 0.95 ^a^	70.23 ± 0.74 ^a^	84.75 ± 0.26 ^a^	16.54 ± 0.34 ^e^	23.03 ± 0.83 ^a^	1.94 ± 0.03 ^b^
US200-CWCS	58.32 ± 0.32 ^f^	67.85 ± 0.54 ^f^	79.68 ± 0.48 ^gh^	19.25 ± 0.62 ^a^	21.36 ± 0.48 ^a^	2.01 ± 0.04 ^a^
US400-CWCS	58.86 ± 0.14 ^e^	68.20 ± 0.97 ^ef^	80.34 ± 0.07 ^g^	18.93 ± 0.19 ^ab^	21.48 ± 0.51 ^a^	2.02 ± 0.05 ^a^
US600-CWCS	59.29 ± 0.25 ^d^	68.64 ± 0.05 ^de^	80.72 ± 0.85 ^f^	18.08 ± 0.21 ^c^	21.43 ± 0.45 ^a^	1.93 ± 0.01 ^bc^
HM15%-US200-CWCS	59.32 ± 0.13 ^d^	68.50 ± 0.38 ^e^	81.18 ± 0.74 ^e^	15.39 ± 0.52 ^ef^	21.93 ± 0.65 ^a^	1.66 ± 0.09 ^c^
HM15%-US600-CWCS	59.60 ± 0.98 ^cd^	68.87 ± 0.44 ^d^	81.73 ± 0.21 ^e^	14.64 ± 0.31 ^g^	22.13 ± 0.28 ^a^	1.57 ± 0.07 ^cd^
HM25%-US200-CWCS	60.07 ± 0.32 ^b^	69.06 ± 0.28 ^b^	82.54 ± 0.39 ^cd^	13.63 ± 0.14 ^h^	22.47 ± 0.38 ^a^	1.51 ± 0.02 ^d^
HM25%-US600-CWCS	60.68 ± 0.54 ^b^	69.43 ± 0.13 ^ab^	82.89 ± 0.94 ^c^	12.18 ± 0.25 ^i^	22.21 ± 0.61 ^a^	1.49 ± 0.03d ^e^

Onset Temperature (T_o_), Peak Temperature (T_p_), Conclusion Temperature (T_c_), Enthalpy of Gelatinization (ΔH), Total Gelatinization Temperature Range (T_r_), and Peak Height Index (PHI). There are significant differences (*p* ≤ 0.05) in the mean ± standard deviation values for each sample in the same column, denoted by different superscripts on the shoulders of the same line. However, non-significant differences (*p* > 0.05) in the mean ± standard deviation values for each solution in the same column are denoted by the same superscripts on the shoulders of the same line.

**Table 3:** The ratios of intensities at wavenumbers 995, 1025, and 1048 cm^−1^ (R_1025/995_ and R_1048/1025_) were measured by FTIR for the native and single-modified (HM, US) and dual-modified (HM-US) Chinese Water Chestnut Starch samples.

Starch Type	R_1025/995_	R_1048/1025_
CWCS	0.52 ± 0.07	1.60 ± 0.02 ^a^
HM15%-CWCS	0.62 ± 0.14 ^d^	1.55 ± 0.17 ^b^
HM20%-CWCS	0.66 ± 0.09 ^cd^	1.46 ± 0.05 ^d^
HM25%-CWCS	0.71 ± 0.06 ^c^	1.38 ± 0.01 ^e^
US200-CWCS	0.60 ± 0.10 ^d^	1.57 ± 0.06 ^b^
US400-CWCS	0.64 ± 0.11 ^cd^	1.50 ± 0.09 ^bc^
US600-CWCS	0.67 ± 0.13 ^c^	1.47 ± 0.13 ^d^
HM15%-US200-CWCS	0.67 ± 0.10 ^c^	1.54 ± 0.07 ^bc^
HM15%-US600-CWCS	0.70 ± 0.04 ^c^	1.45 ± 0.03 ^d^
HM25%-US200-CWCS	0.76 ± 0.02 ^b^	1.36 ± 0.01 ^ef^
HM25%-US600-CWCS	0.79 ± 0.01 ^a^	1.30 ± 0.04 ^f^

There are significant differences (*p* ≤ 0.05) in the mean ± standard deviation values for each sample in the same column, denoted by different superscripts on the shoulders of the same line.

## Data Availability

The original contributions presented in this study are included in the article/Supplementary Materials. Further inquiries can be directed to the corresponding author.

## Supplementary Materials

The following supporting information can be downloaded at: https://www.mdpi.com/article/10.3390/foods14132185/s1, Figure S1: CWCS samples, both native and single modified (HM, US) and dual modified (HM-US), were tested for swelling power and solubility. References [60,61] are cited in Supplementary Materials.
