# Supplementary material for: Implicating Ultrasonication and Heat–Moisture Treatments as a Green and Eco-Friendly Approach for Dual Physical Modification of Eleocharis tuberosa Starch to Improve Its Physico-Chemical and Functional Properties
Source: Foods. 2025 Jun 22;14(13):2185. doi: 10.3390/foods14132185 (PMC12248610; doi:10.3390/foods14132185)
Supplement: Supplementary file 1 [file foods-14-02185-s001.zip › foods-3644852-supplementary.pdf]

Implicating Ultrasonication and Heat-Moisture Treatments as a Green and Eco-Friendly Approach for Dual Physical Modification of *Eleocharis tuberosa* Starch to Improve Its Physico-chemical and Functional Properties

Zafarullah Muhammad [1], Rabia Ramzan [1,2], Chen Ana [1], Muhammad Afzaal [3], Adnan Abbas [4], Muhammad Safiullah Virk [5], Abdullah [6], Wu Sun [1] and Guoqiang Zhang [1,*]

[1] College of Biological and Food Engineering, Anhui Polytechnic University, Wuhu 241000, China;
zafwahla@mail.ahpu.edu.cn (Z.M.); rabiaramzan@mail.ahpu.edu.cn (R.R.); chenana@ahpu.edu.cn (C.A.); wusun1997@163.com (W.S.)
[2] College of Food Science and Technology, Huazhong Agricultural University, Wuhan 430070, China
[3] Department of Food Science, Nutrition and Home Economics, Government College University,
Faisalabad 5400, Pakistan; muhammadafzaal@gcuf.edu.pk
[4] School of Chemical and Environmental Engineering, Anhui Polytechnic University, Wuhu 241000, China; adnanabbas@ahpu.edu.cn
[5] School of Food and Biological Engineering, Jiangsu University, Zhenjiang 212013, China;
safiullahvirk@hotmail.com
[6] College of Food Science and Technology, Zhejiang University of Technology, Hangzhou 310014, China; abdullah_ch2002@yahoo.com
* Correspondence: zhangguoqiang@ahpu.edu.cn; Tel.: +86-0553-2871254

Supplementary materials

1. Methodology

1.1. Solubility and swelling power

The solubility and swelling power of the starch samples were determined using the method described by Y. Zhang et al. (2020) [1]. To create a 2% (w/v) starch solution, 10.00 g (dry basis) of the sample was weighed and dissolved in distilled water. After 30 minutes of gelatinization at 85 °C, each suspension was allowed to cool to room temperature. The weight (P) of swollen starch was then determined by centrifuging the solution for 30 minutes at 3500 rpm and weighing the precipitate. The supernatant was decanted and then dried at 105 °C in an oven until it reached a consistent weight (A). The following equations, S1 and S2, were used to determine the starch samples' S (%) and SP (g/g).

$$S\ (\%) = \frac{A}{W} \times 100 \qquad \text{S1}$$

$$SP\ \left(\frac{g}{g}\right) = \frac{P}{W(100-S)} \qquad \text{S2}$$

2. Discussions

2.1. Solubility and swelling power of native and modified CWCS

Single (HM, US), dual (HM-US), and native CWCS starches were tested for swelling power and solubility at 95 °C (Fig 1S A, B). Compared to HM and US-treated starch, native CWCS starch exhibited a significantly ($p < 0.05$) better swelling power. The CWCS starch's ability to swell may be reduced by ultrasound. The creation of double helices in the US process and the chain depolymerization of starch molecules were the causes of these results. Additionally, when moisture concentrations rose, HM significantly ($p < 0.05$) reduced the swelling power of CWCS starch. HM ascribed the reduction in swelling power to the internal reorganization of lipids and starch chains [2]. Furthermore, CWCS combinedly treated with HM and US significantly ($p<0.05$) decreased starch swelling compared to starches treated with HM alone. Both the internal reorganization of the granules and the amylose leaching via separation and diffusion out of the starch particles during the swelling process were responsible for the starch's solubility in HM treatment [3].

Fig. 1S.

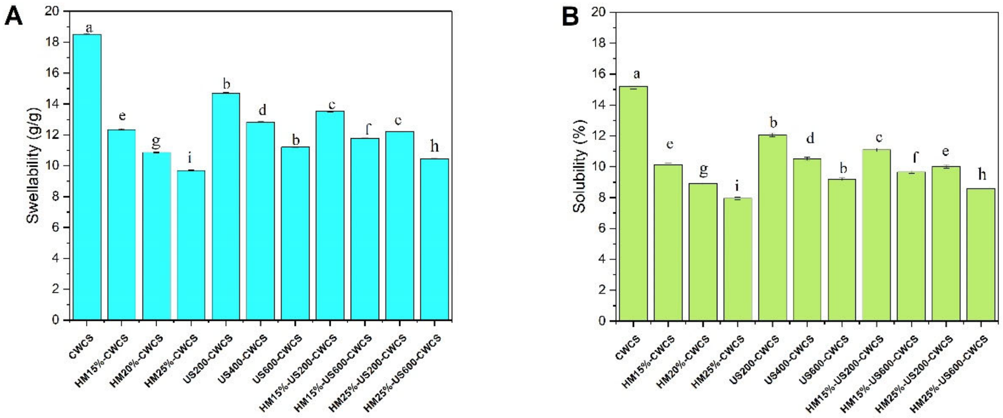

Figure S1. CWCS samples, both native and single modified (HM, US) and dual modified (HM-US), were tested for swelling power and solubility.

References

1. Zhang, Y.; Dai, Y.; Hou, H.; Li, X.; Dong, H.; Wang, W.; Zhang, H. Ultrasound-Assisted Preparation of Octenyl Succinic Anhydride Modified Starch and Its Influence Mechanism on the Quality. *Food Chem X* **2020**, *5*, doi:10.1016/j.fochx.2020.100077.

2. Bharti, I.; Singh, S.; Saxena, D.C. Exploring the Influence of Heat Moisture Treatment on Physicochemical, Pasting, Structural and Morphological Properties of Mango Kernel Starches from Indian Cultivars. *LWT* **2019**, *110*, doi:10.1016/j.lwt.2019.04.082.

3. Han, L.; Wei, Q.; Cao, S.; Yu, Y.; Cao, X.; Chen, W. The Assisting Effects of Ultrasound on the Multiscale Characteristics of Heat-Moisture Treated Starch from Agriophyllum Squarrosum Seeds. *Int J Biol Macromol* **2021**, *187*, doi:10.1016/j.ijbiomac.2021.07.123.
